# Technical Advances in Circulating Cell-Free DNA Detection and Analysis for Personalized Medicine in Patients’ Care

**DOI:** 10.3390/biom14040498

**Published:** 2024-04-19

**Authors:** Monica Sorbini, Tullia Carradori, Gabriele Maria Togliatto, Tiziana Vaisitti, Silvia Deaglio

**Affiliations:** 1 Department of Medical Sciences, University of Turin, 10126 Turin, Italy; tullia.carradori@unito.it (T.C.); tiziana.vaisitti@unito.it (T.V.); silvia.deaglio@unito.it (S.D.); 2 Immunogenetics and Transplant Biology Service, Città della Salute e della Scienza, 10126 Turin, Italy; gabriele.togliatto@unito.it

**Keywords:** cell-free DNA, next-generation sequencing, digital PCR, liquid biopsy, non-invasive diagnostics

## Abstract

Circulating cell-free DNA (cfDNA) refers to small fragments of DNA molecules released after programmed cell death and necrosis in several body fluids such as blood, saliva, urine, and cerebrospinal fluid. The discovery of cfDNA has revolutionized the field of non-invasive diagnostics in the oncologic field, in prenatal testing, and in organ transplantation. Despite the potential of cfDNA and the solid results published in the recent literature, several challenges remain, represented by a low abundance, a need for highly sensitive assays, and analytical issues. In this review, the main technical advances in cfDNA analysis are presented and discussed, with a comprehensive examination of the current available methodologies applied in each field. Considering the potential advantages of cfDNA, this biomarker is increasing its consensus among clinicians, as it allows us to monitor patients’ conditions in an easy and non-invasive way, offering a more personalized care. Nevertheless, cfDNA analysis is still considered a diagnostic marker to be further validated, and very few centers are implementing its analysis in routine diagnostics. As technical improvements are enhancing the performances of cfDNA analysis, its application will transversally improve patients’ quality of life.

## 1. Introduction

In recent decades, the field of molecular biology has experienced a significant step forward with the emergence of circulating cell-free DNA (cfDNA) as a versatile biomarker with relevant clinical implications. CfDNA, consisting of double-stranded DNA fragments released into the bloodstream following cellular apoptosis and necrosis, represents a significant advance in the detection and monitoring of various physiological and pathological conditions. These conditions range from cancer to post-transplant monitoring and prenatal diagnosis, making cfDNA analysis a crucial tool in modern medicine.

This manuscript aims to explore the significance of cfDNA analysis, highlighting its new insights and contributions to the existing literature. By providing a comprehensive overview of its diverse origins, clinical applications, and technical challenges, it aims to serve as a contribution in the understanding and use of cfDNA as a biomarker.

At its core, cfDNA analysis represents a non-invasive and innovative approach, often referred to as “liquid biopsy”. This term underscores its ability to provide diagnostic and prognostic information by analyzing genetic material obtained from bodily fluids, primarily blood plasma or serum. This revolutionary technique holds significant promise in various fields, including oncology, prenatal screening, and transplantation medicine.

Through a detailed examination of recent advancements, this review aims to clarify the crucial roles of cfDNA in disease detection, treatment monitoring, and patient management.

Through a structured analysis of key themes and methodologies, this review aims to serve as a valuable resource for researchers, clinicians, and healthcare professionals engaged in cfDNA analysis.

By providing insights into the evolving landscape of cfDNA research and its potential implications for personalized medicine, this manuscript is a valuable resource for researchers, clinicians, and healthcare professionals engaged in cfDNA analysis. Ultimately, by advancing our understanding of cfDNA and its clinical applications, this manuscript contributes to the ongoing dialogue on this revolutionary biomarker, paving the way for better diagnostic and therapeutic strategies in modern medicine.

## 2. Circulating Cell-Free DNA

CfDNA is represented by double-stranded extracellular DNA fragments released into the bloodstream after the apoptosis and necrosis processes in physiological and pathological situations. It was first described in 1948 [1] when Mandel and Matais detected the presence of DNA in plasma samples from healthy and affected individuals. CfDNA originates from many sources within the body and can be isolated from various body fluids such as blood, urine, effusions, and cerebrospinal fluid [2]. In healthy conditions, it derives mainly from blood cells [3,4], but it can arise from inflammatory cells, tumor cells, fetal cells crossing the placenta during pregnancy, or can be released from graft cells after solid organ transplantation [5]. Human plasma DNA consists of a mixture of DNA fragments of different sizes, mostly ranging between 100 and 200 base pairs [6,7], with a peak at 166 bases; this peculiar length was related to the nucleosomal structure [8,9], as during the cell death process, proteins associated with DNA seem to protect short fragments from degradation. However, smaller (<100 bases) or larger fragments of several kilobases have also been reported [10,11,12] and associated, respectively, to mitochondrial and necrotic origin [9,13]. The CfDNA concentration in blood widely ranges between undetectable and a high concentration (up to 100 ng/mL) in healthy subjects [9,13], but it is known that its levels can be affected by many individual conditions, such as age, BMI, circadian rhythm [13], exercise [4], inflammation [5], infections [14,15,16], and pharmacologic treatment [5], that tend to increase cfDNA presence. 

## 3. cfDNA Applications in Clinical Care

Since the discovery of cfDNA, its potential applications in various fields have been continuously explored. The application of cfDNA analysis, which is defined as “liquid biopsy”, is used to monitor pathological conditions in oncologic, prenatal, and transplantation fields in a non-invasive and revolutionary method [9].

### 3.1. Oncologic Applications

In oncology, the presence of the circulating tumor cfDNA (ctDNA) and the analysis of its genetic alterations allows the detection of cancer disease, the monitoring of treatment response, and the detection of minimal residual disease, enabling personalized treatment strategies [17,18]. Currently, the most common use of ctDNA analysis is therapy selection and stratification of patients based on the likelihood of response to targeted therapies [19,20,21,22] by searching for specific mutation markers for resistance or sensitivity, such as tyrosine kinase inhibitors, programmed death inhibitors-1 [23], programmed death ligand-1 [24], and cytotoxic T lymphocyte-associated protein 4 [25]. Through ctDNA analysis, it is, therefore, possible to differentiate and predict immune checkpoint blockade response patterns [26,27], characterize the tumor heterogeneity [28], and detect resistance for targeted therapy and chemotherapy early [29,30,31,32].

Another important and recent use of ctDNA is the approximation of tumor burden [33,34] enabled as the ctDNA quantity is directly associated with the number of tumor cells present in the body.

Methylation markers have also been proposed for the detection of early cancer, with the advantage of discriminating the tissue of origin of the cfDNA based on the tissue-specific methylation pattern [35,36]. 

### 3.2. Prenatal Screening

In prenatal testing, the analysis of fetal cfDNA in maternal blood has revolutionized the field, allowing non-invasive prenatal testing (NIPT) that can investigate chromosomal abnormalities and fetal aneuploidies as an alternative to more invasive methods such as karyotyping and FISH on fetal blood, chorionic villus sampling, or amniocentesis [37]. Non-invasive prenatal screening can be performed from 5 to 7 weeks [38], looking for pathological variations with a targeted or genome-wide approach. In addition, it is possible to noninvasively determine fetal sex, genotype fetal blood group D antigen, and detect variants involved in paternally inherited or de novo disorders [39].

To date, a low fetal fraction is the most important cause of negative results in cfDNA screening and is reported as a cause of test failure in up to 6.1% of tests performed [40,41]. This poor fetal cfDNA concentration may result from an increased maternal fraction due to the conditions of the mother [4,13], both physiological, such as intense physical exercise, age, BMI, or the circadian rhythm, and pathological, such as in the case of concomitant inflammation [5] or infection [14,15,16]. All these conditions tend to cause a stronger release of background DNA from the mother, resulting in an apparent failure to detect the fetal DNA fraction and in the test’s failure.

### 3.3. Transplantation

Clinical studies have highlighted the potential of detecting and quantifying the fraction of donor-derived cell-free DNA (dd-cfDNA), i.e., the portion of cfDNA derived from the transplanted organ, to monitor transplant status and detect rejection earlier and with greater sensitivity than traditional methods, such as graft biopsy, allowing early intervention and improved transplantation outcomes [42,43,44]. Dd-cfDNA has been shown to be a potential biomarker of acute rejection, well correlating with biopsy-proven rejection, and more generally, it is a signal of graft damage, post-transplant complications, and infection. Differences in the percentage of dd-cfDNA between graft types have been observed, reflecting the effective size and the organ-specific cell turnover [45], similar to results reported for ctDNA changes associated with tumor burden.

Dd-cfDNA is discriminated from recipient cfDNA by exploiting widespread genetic polymorphisms in the genome. The first published approaches to detect dd-cfDNA relied on a panel of short-tandem repeats (STRs), variable-number tandem repeats (VNTRs), single nucleotide (SNPs), or insertion–deletion polymorphisms (INDELs) chosen as polymorphic enough to distinguish all possible donor–recipient pairs and therefore were defined as “targeted approaches” [46,47] as they target pre-selected sequences in the genome. A particular method to discriminate the portion of dd-cfDNA present in the bloodstream is based on the donor and recipient Human Leukocyte Antigen (HLA) typing [48,49]. Since transplant centers generally check the HLA loci to identify the best match for transplantation, this information is therefore available and can be used to discriminate donor cfDNA from that of the recipient.

More recent NGS techniques do not require genotyping and are commonly called “random approaches” since after the sequencing phase, specific donor, and recipient polymorphisms are selected based on the genomic profile of both subjects [50,51].

The identification of cfDNA tissue source may represent a valid alternative for graft versus host disease (GVHD) non-invasive detection. Acute GVHD remains an important complication after allogeneic hematopoietic cell transplantation (HCT) [52]. Currently, there are no validated non-invasive biomarkers that are used in routine clinical applications for acute GVHD. Candidate molecules were cytokines and peptides involved in the systemic inflammation and pathophysiology of GVHD, but their performance resulted in limited and poorly specific [52]. As the liver, skin, and intestine are the most involved organs in the disease, a significant increase in cfDNA deriving from these tissues can be informative of the development of the pathology [53].

Each tissue is characterized by an epigenetic signature that allows for the identification of the DNA origin through the analysis of its methylation profile [54]. Advanced molecular analyses as whole-genome bisulfite sequencing allow for the correct identification and quantification of the cfDNA source, enabling the non-invasive monitoring of GVHD [55]. This approach has been tested by Pellan Cheng and colleagues [56], who analyzed a pilot cohort of HCT recipients, and the result of their proof-of-principle study showed the potential of cfDNA to assist in personalizing care after HCT.

## 4. Technical Issues for High-Quality cfDNA Analysis

### 4.1. The Relevance of Correct Sampling

Performing a liquid biopsy means, in practice, the retrieving of cfDNA from a body fluid, mostly peripheral blood. However, the rapid turnover and short half-life of cfDNA [9,13] require proper sampling, considering the relatively low concentration of this marker. Most studies were performed using EDTA BD vacutainer [57,58], which does not preserve blood cells from apoptosis and release genomic DNA, affecting the quantity and quality of cfDNA itself [16,59] if the plasma is not rapidly separated from the corpuscular part [13]. To prevent cfDNA degradation and its dilution into genomic DNA, ad hoc collection tubes are available from different companies (Qiagen, Hilden, Germany, Roche, Basel, Switzerland, and Streck, La Vista, NE, USA), which were successfully used in some studies [60,61]. Their main advantage is that tubes keep cfDNA stable and free from genomic contamination for up to 14 days, improving the performance of the following research studies, drug discovery, and assay development.

To improve the purity of cfDNA, it is essential to effectively separate plasma from other blood fractions containing cells which can potentially contaminate the sample. A two-step centrifugation procedure is commonly employed when working with cfDNA, as it allows for the removal of cellular debris still present in the plasma specimen after the initial centrifugation [13]. However, the recommended centrifugation protocol is typically provided in the tube datasheet.

Additionally, the selection of the appropriate extraction method is crucial for ensuring high-quality cfDNA. The majority of commercial kits for cfDNA analysis recommend the use of validated options to achieve optimal results in terms of both quantity and purity of the cfDNA. Among the plethora of available extraction kits, a significant proportion involve capturing cfDNA fragments using magnetic beads to separate them from genomic DNA contaminants, followed by repeated washing steps to effectively isolate nucleotides from proteins and lipids.

Nevertheless, prior to conducting molecular tests, it is highly advisable to precisely quantify the samples using a fluorometric instrument and verify the expected fragment size using automated electrophoresis systems. These systems can readily detect genomic contamination by analyzing a small volume of cfDNA.

### 4.2. Technical Comparison of cfDNA Analysis Methods 

**Figure 1:**
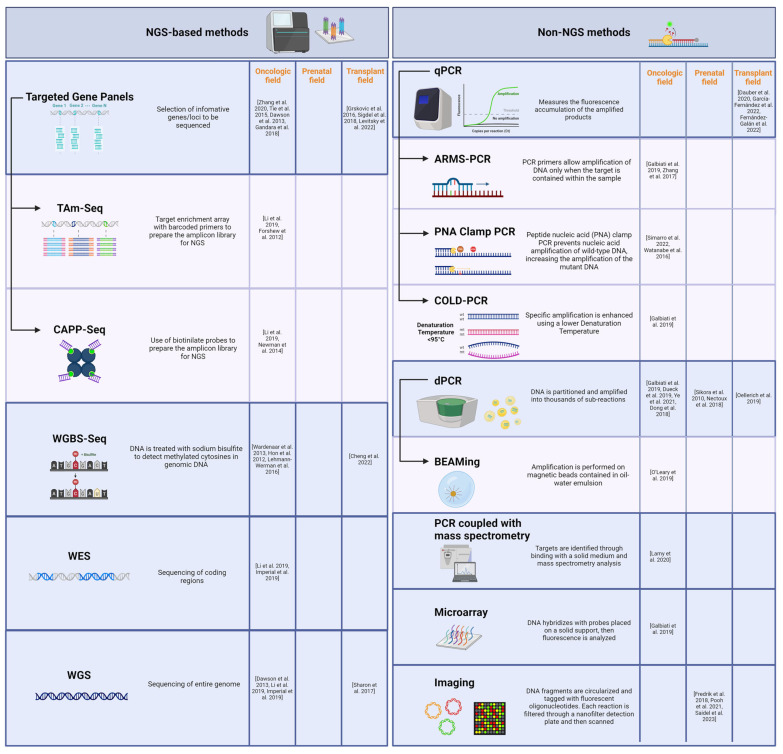
List of the NGS-based and non-NGS methods for cfDNA analysis described in the review. The different methodologies are divided according to their technological approaches. The main methods are highlighted in blue, while derived methods are indicated by arrows. References are listed by application field [27,29,33,34,35,39,46,47,51,56,57,62,63,64,65,66,67,68,69,70,71,72,73,74,75,76,77,78,79,80,81,82,83,84]. NGS: Next-generation Sequencing; TAm-Seq: Tagged-amplicon Deep Sequencing; CAPP-Seq: Cancer Personalized Profiling by Deep Sequencing; WGBS-Seq: Whole Genome Bisulfite Sequencing; WES: Whole Exome Sequencing; WGS: Whole Genome Sequencing; qPCR: quantitative PCR; ARMS-PCR: Amplification Refractory Mutation System PCR; PNA Clamp PCR: Peptide Nucleic Acid Clamp PCR; COLD-PCR: Co-amplification at Lower Denaturation Temperature-based PCR; dPCR: digital PCR; BEAMing: Beads, Emulsion, Amplification, Magnetics PCR.

Advancements in technology, particularly the advent of quantitative PCR (qPCR) and next-generation sequencing (NGS), significantly enhanced the detection sensitivity and precision of cfDNA analysis. Methods for cfDNA analysis are generally divided into NGS and non-NGS approaches (Figure 1).

#### 4.2.1. NGS-Based Methods

NGS-based approaches have the potential to simultaneously sequence thousands of targets. Considering the Illumina technology, its high accuracy and flexibility made it the most spread platform for cfDNA analysis compared to competitors, such as Ion Torrent, Oxford Nanopore, and Pacific Biosciences, which are still limited by their technical features that do not apply properly with short cfDNA fragments [85,86].

In the NGS workflow, DNA samples are amplified targeting hundreds or thousands of single nucleotide polymorphisms (SNPs) [33,46,87,88] selected depending on the application field, then DNA fragments are tagged by adaptors and indexed before being sequenced with an elevated depth that permits sensitive results after bioinformatics analyses. Assay types can vary according to the aim of the analysis, moving from tagged-amplicon deep sequencing (TAm-Seq), if the target sequence has been previously characterized [62,63], to personalized profiling by deep sequencing, such as CAPP-Seq applied in oncology [62,64], to whole genome bisulfite sequencing (WGBS-Seq) for DNA methylation analysis [65,66], and to whole exome (WES) or genome sequencing (WGS), which provide a comprehensive evaluation of tumor mutations, identifying potential oncogenes and tumor suppressor genes, deleterious alterations, and variants of unknown significance [62,67]. However, WES and WGS are limited by low sensitivity, excessive time and cost, and difficulties in the interpretation of results [2].

For accurate detection of low-abundance targets, such as in the case of liquid biopsy in which the fraction of target DNA within a cfDNA sample is potentially poorly represented, deep sequencing is necessary to provide the required sensitivity [89]. Recent improvements in sequencing instrumentation offer options with extremely high coverage depth for large portions of the entire genome in a single sample [90]. Although the cost of performing NGS has decreased considerably [91], this method can have a relatively consistent cost with a long turnaround time (often at least 3 days) and with variable sensitivity. Indeed, when assays are designed to cover several genetic targets, the comprehensive nature of NGS can provide value in efficiency and cost reduction, while NGS is more expensive and time-consuming when analyzing a small number of variants or samples [92]. Moreover, NGS does not always provide an absolute quantification of cfDNA meant as the total number of DNA copies [42,43,44,50,93,94,95,96,97]. 

#### 4.2.2. Non-NGS Methods

Real-time or qPCR, microarrays, and digital PCR (dPCR) are included in non-NGS methods and offer a faster and less expensive detection option compared to NGS. These methods are generally used to detect and quantify the presence of known specific mutations or polymorphisms in cfDNA samples [14,68,69,70,71]. However, to enhance assay sensitivity, improved PCR approaches were developed. To identify single base changes or short deletion, the amplification refractory mutation system (ARMS-PCR) exploits sequence-specific PCR primers that allow amplification of DNA only when the target is contained within the sample, thus lowering the limit of detection in comparison with conventional PCR [71,72]. The same results can be obtained by peptide nucleic acid (PNA) clamp PCR, which prevents the nucleic acid amplification of wild-type DNA, increasing the amplification of the mutant DNA [73,74]. Another alternative is the co-amplification at lower denaturation temperature-based PCR (COLD-PCR), which results in the enhancement of both known and unknown minority alleles during PCR, irrespective of the mutation type and position. This method is based on the exploitation of the critical temperature at which mutation-containing DNA is preferentially melted over the wild type [71].

To increase the number of targets that can be examined simultaneously, PCR can be coupled with mass spectrometry. After amplification, PCR products are analyzed with mass spectrometry, searching for dozens of target mutations in a single reaction with great sensitivity [75].

Besides encouraging results, qPCR efficiency may be affected by variations in amplification. Furthermore, qPCR measures the fluorescence accumulation of the amplified product and requires normalization to a standard curve or to a reference, resulting in a relative quantification. The main difference between qPCR and dPCR is that, unlike conventional amplification, the reaction in dPCR is partitioned into thousands of sub-reactions, allowing absolute quantitation and high sensitivity. DPCR was first described in 1992 by Sykes et al., who changed standard amplification with the integration of limiting dilution, end-point PCR, and Poisson statistics [98]. While partitioning the samples in thousands of independent amplification reactions, dPCR reach higher accuracy and an absolute quantification of the target, which is determined by Poisson statistics. The evolution of the Sykes method was achieved by Vogelstein and Kinzler who added the detection of the target through fluorescent probes to the partitioning of the sample [99]. Current dPCR technology uses reagents and workflows similar to those used for most standard TaqMan probe-based assays with a smaller sample requirement, reducing cost and preserving precious samples. The methods described by Sykes, Vogelstein, and Kinzler have been improved and are commercially available as different platforms. dPCR amplification can be performed on a microfluidic chip [100], microarrays [101], or spinning microfluidic discs [102] or can be based on oil–water emulsions [76]. Moreover, dPCR technology enables high-throughput analysis with a reduced cost compared with other methods while maintaining great sensitivity and accuracy. Moreover, because cfDNA is poorly concentrated in plasma, repeated testing on different sample aliquots may not be possible. DPCR can overcome this limit, since it allows for an accurate detection and quantitation without separate calibration reactions [103], resulting in a reagent and sample saving. Compared with commercial qPCR assays [94], dPCR assays achieve a better limit of detection as well as a more accurate result.

However, dPCR shows practical drawbacks. The number of targets that can be detected is significantly lower compared to NGS-based methods due to the possibility of a multiplex from two to a maximum of six fluorophores using the most innovative instruments. Moreover, limitations in droplet-to-droplet volume uniformity can influence the quantification accuracy and reproducibility, but fluidics-based dPCR may offer an opportunity to overcome this limitation [77,104]. Then, PCR efficiency can vary due to different amplicon lengths [105], as longer amplicons are amplified less efficiently, which might result in underestimation of the true cfDNA value [78]. Similarly, Dauber et al. demonstrated that the cfDNA concentration was five times higher when using smaller amplicons compared with larger amplicons [68]. Therefore, the use of short amplicons is recommended for the accurate quantification of cfDNA to avoid underestimation of the target.

NGS and dPCR techniques were demonstrated to produce similar results in different application fields. The comparison on kidney transplant recipient samples highlighted no significant differences in the detection of cfDNA, with a significant association between the measurements obtained with both methods [106]. Moreover, lower limits of quantification were similar and in line with what is already reported in the literature [107], even though NGS method resulted more sensitive in the lower range than the dPCR method [106]. The quantification of mixed chimerism after hematopoietic stem cell transplantation appeared to be feasible with both methodologies conserving high performances in terms of sensitivity, reproducibility, and linearity [108]. Conversely, dPCR performed better in the detection of *KRAS* mutation in the oncologic field, with high sensitivity and specificity [79], and a limit of quantification 10-fold lower compared to NGS [80].

A great advantage of dPCR is the possibility to obtain the absolute concentration of the target, expressed as copies/µL or copies/mL, which is not influenced by fluctuations in the background cfDNA, derived from the patient. Indeed, NGS results can be expressed only in a cfDNA percentage that can be biased and underestimated as a consequence of physiological or pathological conditions of the subject (e.g., concomitant infections, BMI, exercise, etc.) [4,5,13,14,15,16]. The use of cfDNA as a concentration has also been shown to be superior to the ratio as a biomarker for allograft rejection [81].

In contrast with amplification-based methods, an imaging single-DNA-molecules method for high-precision cfDNA detection was developed. In the VANADIS assay (PerkinElmer, Waltham, MA), DNA fragments are labeled with fluorescent oligonucleotides specific for precise genetic targets, then circularized and copied multiple times before being placed on a 96-well nanofilter microplate and analyzed by imaging [82]. This assay is now applied to prenatal screening with high accuracy [83,84]. Since this method does not require DNA amplification and sequencing, it is easily implemented in any laboratory, scalable, and fully automated.

## 5. Conclusions

Given the potential applications of cfDNA, this biomarker is increasing the general agreement among clinicians in the oncology, prenatal, and transplantation fields. Despite the encouraging results, however, the cfDNA analysis is not a reality as it is exploited in a relatively small number of centers, and it is still considered a research marker to be further validated. Challenges persist in cfDNA analysis, highlighted by the scarcity of biomarkers, the necessity for developing highly sensitive methodologies, and the analytical complexities linked to processing and interpretation. Nevertheless, ongoing research endeavors strive to overcome these obstacles, potentially enhancing the clinical utility of cfDNA and facilitating its integration into routine diagnostic practices.

Novel and more powerful technologies are improving the sensitivity and the performances of cfDNA analysis, making its application easy, feasible, and attracting. NGS and dPCR, which are the main players in liquid biopsy, serve distinct purposes. NGS is a powerful tool for large-scale sequencing and genomics studies, while dPCR excels in quantifying specific cfDNA targets with exceptional precision and sensitivity. Considering the costs, NGS can be cost-effective for high-throughput sequencing projects but may be expensive for small-scale studies, while dPCR is generally more cost-effective for targeted, low-throughput applications. Therefore, the choice between these techniques should be based on the specific research goals and the scale of the project.

It is true that currently it is not entirely clear whether cfDNA can be considered a reliable diagnostic tool compared to standard methods. Regulations and regulatory agencies may vary significantly from country to country, which can influence the adoption and use of cfDNA in clinical practice [109,110].

In addition, the lack of standardized guidelines for the validation and reporting of cfDNA methods is a significant challenge in the field. While some progress has been made in prenatal diagnosis, there remains a need for comprehensive guidelines across various applications of cfDNA analysis [111]. Establishing such guidelines would not only enhance the reliability and reproducibility of cfDNA-based tests but also facilitate their wider adoption in clinical practice.

However, there is growing evidence suggesting the potential of cfDNA as a diagnostic marker in various clinical contexts, such as oncology, prenatal screening, and transplantation medicine. While further research and validation of cfDNA as a diagnostic tool are needed, advances in technology and understanding of cfDNA biology are contributing to making it increasingly promising as an integral part of disease diagnosis and monitoring. With additional studies and international collaborations, we may be able to better clarify the role and efficacy of cfDNA in different clinical settings and harmonize regulations to promote uniform adoption of this innovative technology.

In conclusion, the introduction of liquid biopsy offers new insights into disease detection and treatment response monitoring in the evolving field of precision medicine. In the future, cfDNA could be applied transversely to achieve a more personalized medicine, improving patients’ quality of life.
